# Biotic vs. Abiotic Control of Decomposition: A Comparison of the Effects of Simulated Extinctions and Changes in Temperature

**DOI:** 10.1371/journal.pone.0087426

**Published:** 2014-01-23

**Authors:** Luz Boyero, Bradley J. Cardinale, Mikis Bastian, Richard G. Pearson

**Affiliations:** 1 Wetland Ecology Department, Doñana Biological Station-CSIC, Sevilla, Spain; 2 School of Marine and Tropical Biology, James Cook University, Townsville, Queensland, Australia; 3 School of Natural Resources and Environment, University of Michigan, Ann Arbor, Michigan, United States of America; 4 TropWater, James Cook University, Townsville, Queensland, Australia; Institute of Botany, Czech Academy of Sciences, Czech Republic

## Abstract

The loss of species is known to have significant effects on ecosystem functioning, but only recently has it been recognized that species loss might rival the effects of other forms of environmental change on ecosystem processes. There is a need for experimental studies that explicitly manipulate species richness and environmental factors concurrently to determine their relative impacts on key ecosystem processes such as plant litter decomposition. It is crucial to understand what factors affect the rate of plant litter decomposition and the relative magnitude of such effects because the rate at which plant litter is lost and transformed to other forms of organic and inorganic carbon determines the capacity for carbon storage in ecosystems and the rate at which greenhouse gasses such as carbon dioxide are outgassed. Here we compared how an increase in water temperature of 5°C and loss of detritivorous invertebrate and plant litter species affect decomposition rates in a laboratory experiment simulating stream conditions. Like some prior studies, we found that species identity, rather than species richness per se, is a key driver of decomposition, but additionally we showed that the loss of particular species can equal or exceed temperature change in its impact on decomposition. Our results indicate that the loss of particular species can be as important a driver of decomposition as substantial temperature change, but also that predicting the relative consequences of species loss and other forms of environmental change on decomposition requires knowledge of assemblages and their constituent species' ecology and ecophysiology.

## Introduction

The loss of species is known to have significant effects on ecosystem processes [1]–[3], but until recently the magnitude of such effects has not been regarded as sufficient to rival other forms of environmental change that are altering ecosystem functioning globally [4]. The results of two recent syntheses [5], [6] suggest that the impact of changes in biodiversity on key ecosystem functions like productivity and decomposition are as large as other forms of environmental change. However, one of these syntheses [6] focused on a comparison of various experimental manipulations performed in a single grassland ecosystem, while the other [5] compared studies that were performed with entirely different organisms, at divergent scales. Consequently, it is hard to know how broadly the conclusions of these studies apply. To complement such syntheses, we need experimental studies that manipulate species richness and other forms of environmental change concurrently to determine their relative impacts on the same ecosystem processes.

Several studies have manipulated species richness in factorial combination with environmental variables, but most have focused on plant biomass production [7], [8]. There are fewer reported factorial manipulations for other processes, such as plant litter decomposition in fresh waters (but see [9],[10]), even though decomposition is among the most important ecosystem processes in the biosphere [11]. Terrestrial plants produce c. 120 billion tons of organic carbon each year [12], but only a small fraction of it is removed by herbivores [13], while up to 90% enters the pool of dead organic matter [11]. The rate at which plant litter is lost and transformed to other forms of organic and inorganic carbon determines both the capacity for carbon storage in ecosystems, and the rate at which greenhouse gasses such as carbon dioxide (CO_2_) are outgassed, which in turn may alter the climate [14]. It is thus crucial to understand what factors affect the rate of plant litter decomposition and the relative magnitude of such effects.

Temperature is the most obvious of the factors that influence decomposition. Metabolic rates generally increase exponentially with temperature [15], which suggests that decomposition should be highly sensitive to even small changes in temperature [16]–[18]. Biodiversity has also been shown to affect decomposition, but effects are generally weaker than biodiversity effects on plant biomass production [19]. This is particularly true for bottom-up effects – i.e., those driven by plant litter species richness – compared to top-down effects, driven by detritivore species richness [20].

Few studies have examined the effects of temperature and detritivore species richness on decomposition simultaneously (but see [21]), and none has examined the effects of temperature and both plant litter and detritivore species richness on decomposition. In this study we experimentally manipulated both plant litter and detritivore species richness in factorial combination with temperature to test the null hypothesis that the consequences of losing detritivore or plant litter species are no different from the consequences of increasing temperature, over a selected temperature range. We measured detritivore-mediated decomposition rates in a laboratory experiment, using plant litter and functionally similar leaf-shredding detritivores from an Australian stream. Our design included two levels of plant litter and detritivore species richness (one vs. three species) and two temperatures: the mean stream temperature at the time of animal collection (15°C) vs. a 5°C increase, which falls within the upper confidence interval of the A2 scenario within IPCC predictions for 2100 [22]. We were interested in both the magnitude and direction of any effects, including possible additive or synergistic effects, and in whether richness per se or species identity was the important factor in any significant relationship. From previous work we expected a 5°C increase to cause an increase in processing of about 50% (Nolen and Pearson 1993).

## Materials and Methods

### Organisms and experimental set-up

We conducted an experiment in April-August 2005 using a leaf-shredding detritivore assemblage from a tropical stream in north-eastern Australia (Little Birthday Creek, 18.97°S 146.17°E, 850 m asl). We used the 3 dominant detritivore species, all of them cased caddisfly larvae: *Anisocentropus kirramus* (Calamoceratidae), *Lectrides varians* (Leptoceridae) and *Triplectides gonetalus* (Leptoceridae) [23]. Detritivore treatments included each of the 3 monocultures and the 3-species polyculture. Animals were hand-collected from the stream substrate and acclimated in containers with stream water at stream temperature (15°C) for 3 days, during which they were fed ad libitum with a mixture of leaves other than those used in the experiment (mainly *Eleaocarpus* spp., *Sloanea* spp. and *Abrophyllum ornans*). Collecting permits were provided by the Queensland Department of Environment and Resource Management.

Plant litter provided to detritivores consisted of leaf pieces of common riparian tree species, which varied in specific leaf area (SLA  =  ratio of leaf area to leaf dry weight), a measure that correlates well with decomposition [24]. We compared single-species treatments (3 leaf pieces per replicate) of buff alder *Apodytes brachystylis* (SLA  = 128±30 SD cm^2^ g^−1^ dry weight), the laurel *Cryptocarya leucophylla* (SLA  = 100±10), and blush walnut *Endiandra bessaphila* (SLA  = 77±4), to the 3-species polyculture (1 leaf piece of each species). Undamaged leaves of similar size were collected from the tree (green leaves are prominent in tropical Australian streams [25]), all from a similar height and the same side of the tree. Leaf pieces of similar size with no major veins were cut, air-dried to constant weight, weighed (mean ± SD initial dry weight  = 0.20±0.01 g in all cases), and conditioned in tap water for 48 h prior to the experiment. We used 20 additional leaves of each species to measure SLA; we scanned the leaves, estimated their area with ImageJ 10.2 for MacIntosh, dried them to constant weight and weighed them.

The experiment was run in a temperature-controlled room with a natural (12∶12 h) light:dark photoperiod simulating conditions of heavy shade, with water temperature at 15°C or 20°C (±0.5°C). The lower temperature was chosen because it was the average stream temperature at the time of detritivore collection; the higher temperature simulated a large mean global temperature increase that is possible during this century [22], but was within normal bounds for the study species. Each experimental unit was a 25×11×8 cm plastic container filled with a mixture of stream water and dechlorinated tap water (50∶50%), and leaf pieces of 1 or 3 species. Six caddisfly larvae of similar size (within species) were added to each treatment container, with either 6 larvae of the same species (*A. kirramus*, *L. varians* or *T. gonetalus* only), or 2 larvae of each of the 3 species; control containers had leaves (same as treatment containers) but no detritivores. We ran 3 experimental trials at each temperature (15°C or 20°C); trials were run in random order. Each trial had 2 replicates of each leaf/detritivore combination; we thus had a total of 192 experimental units: 4 leaf treatments x 4 detritivore treatments x 2 temperature treatments x 3 trials x 2 replicates. The experiment lasted for 10 days, after which animals and remaining leaf material were dried at 50°C for 48 h and weighed.

### Data analysis

Detritivore-mediated decomposition rates were quantified as the proportion of leaf dry weight loss, corrected by leaf weight loss in controls to account for effects of microbial decomposition. We calculated decomposition rates per capita and per mg of detritivore; the latter were logit-transformed to attain normality and equal variances (tested with Shapiro-Wilk and Levene's tests, respectively). We present rates per capita as our main results and per mg of detritivore as Figure S1.

To explore variation in decomposition rates, we used a general linear model (GLM) that included all factors (detritivore assemblage, plant litter assemblage and temperature) and their interactions, as well as a covariate to account for temporal variation in the experiment (trial, nested within temperature). Because the detritivore and plant assemblages were significant, we further explored the differences between different assemblages. Firstly, we used post-hoc Tukey tests to assess differences among pairs of assemblages. Secondly, we used a GLM to separate the effects of species richness (1 vs. 3 species) from those of species identity [26]; this model had two nested factors: detritivore species (nested within detritivore species richness), and plant litter species (nested within plant litter species richness). Thirdly, we further explored detritivore and plant species identity effects with a GLM where factors were the presence/absence of each species [27]. Finally, we compared the magnitude of the difference in decomposition rates between the detritivore/plant litter polyculture and each monoculture, as well as between high and low temperature treatments, through effect sizes (Cohen's *d*) and 95% confidence intervals, using the tool provided in www.campbellcollaboration.org/resources/effect_size_input.php. When effects of losing detritivore or plant litter species were significant, we tested whether these effects were significantly different from temperature effects. We considered that two effects were significantly different at the 0.05 level when the difference between the means exceeded twice its standard error, that is, when the following equation was true: mean_B_ – mean_A_ >2 √(SE_A_^2^ + SE_B_^2^) [28].

Because we found no net effect of detritivore species richness on decomposition, but we did find differences based on the presence or absence of different species, we hypothesized that these differences could be driven by the different body sizes of the different species. Size is an important species trait that affects consumption rates but has been ignored in most studies of biodiversity effects on ecosystem functioning in fresh waters [29].We examined body-size effects on decomposition in several ways. Firstly, we examined differences in body size (measured as dry weight of each individual in a replicate) between different detritivore assemblages with a GLM and post hoc Tukey tests. We then used linear regression to examine the relationship between body size and decomposition rates. Finally, we compared the effect size of body size to the effect size of temperature on decomposition rates; for that purpose, we converted the correlation index *r* derived from linear regression analysis to Cohen's *d* using the formula *d* = 2*r*/√1−*r* ^2^ [30].

## Results

### Detritivore species loss vs. temperature

**Figure 1:**
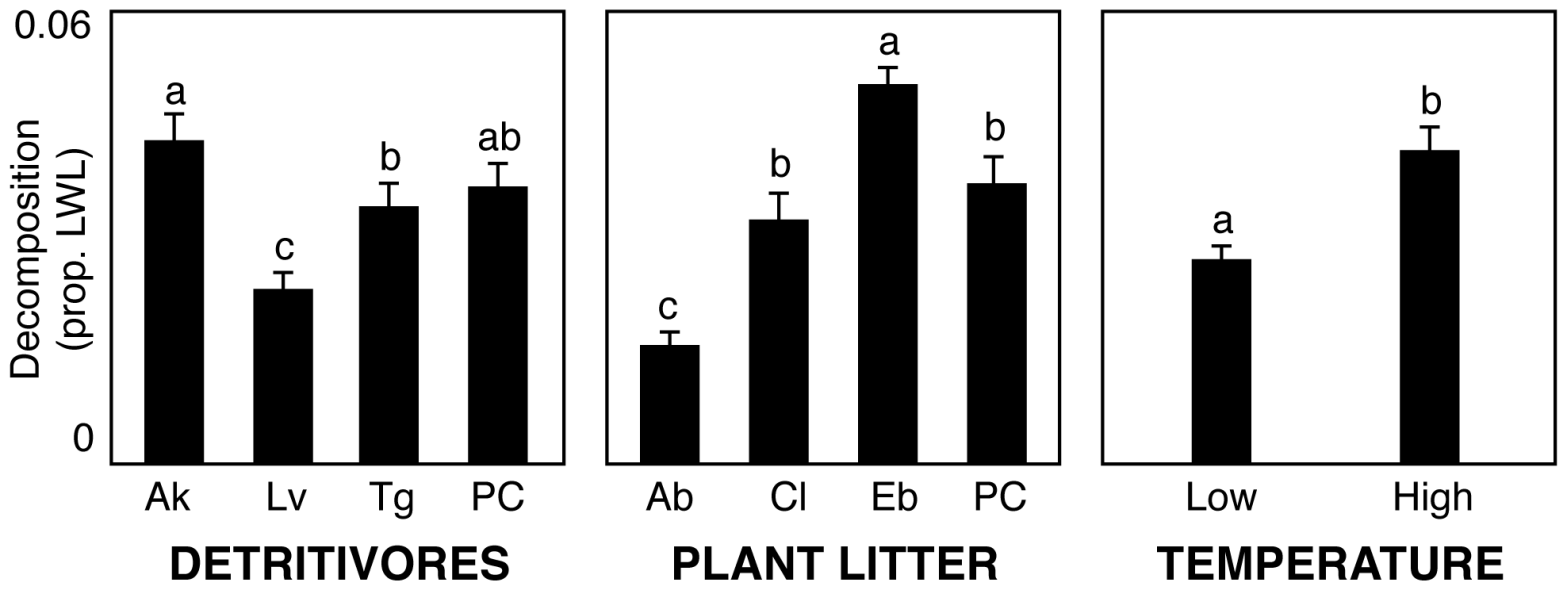
Decomposition rates. Mean ± SE detritivore-mediated decomposition rates (measured as the proportion of leaf weight loss per capita) in each detritivore/plant litter assemblage and temperature treatment. Different letters within panels indicate significant differences (Tukey test, α = 0.5). Ak, *Anisocentropus kirramus*; Lv, *Lectrides varians*; Tg, *Triplectides gonetalus*; Ab, *Apodytes brachystyllis*; Eb, *Endiandra bessaphila*; Cl, *Cryptocarya leucophylla*; PC, polyculture.

**Figure 2:**
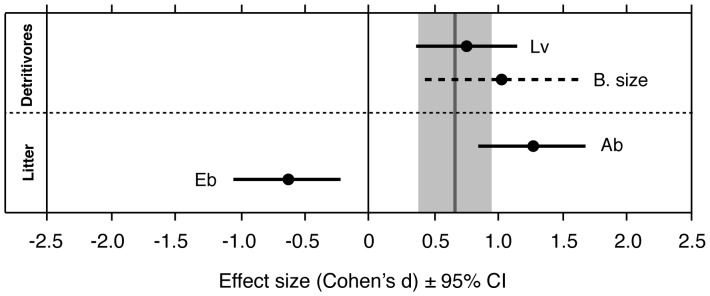
Effect sizes. Effect sizes (Cohen's *d* and 95% confidence interval) of detritivore and plant litter assemblages (each monoculture compared to the polyculture; solid bars) and detritivore body size (broken bars) compared to temperature (grey shade), on detritivore-mediated decomposition rates (measured as the proportion of leaf weight loss per capita). Only significant effects are shown. B. size, detritivore body size; Lv, *Lectrides varians*; Ab, *Apodytes brachystyllis*; Eb, *Endiandra bessaphila*.

There were significant effects of the detritivore assemblage on decomposition (Table 1). However, these effects were due to differences in decomposition between the different monocultures, rather than differences due to species richness per se (Table 1, Fig. 1). Different detritivore species had different effects on decomposition (Table 2): the presence of *A. kirramus* in a treatment resulted in faster decomposition than in treatments without it, *L. varians* had the opposite effect, and *T. gonetalus* had no effect. The analysis of effect sizes partly confirmed these results (Fig. 2): while there was no significant effect when the detritivore polyculture was compared to the mean monoculture, the effect of comparing the polyculture to the *L. varians* monoculture was significant.

**Table 1:** Results of general linear models testing the effects of detritivore and plant litter assemblages and water temperature on detritivore-mediated decomposition rates (measured as the proportion of leaf weight loss per capita).

Source	df	SS	*F*	P
**Model I**				
D	3	0.0099	12.62	<0.0001
L	3	0.0288	36.60	<0.0001
T	1	0.0102	39.11	<0.0001
D x L	9	0.0055	2.34	0.02
D x T	3	0.0009	1.14	0.34
L x T	3	0.0013	1.63	0.18
D x L x T	9	0.0016	0.66	0.74
Trial	4	0.0011	1.01	0.41
Error	184	0.0408		
**Model II**				
DR	1	0.0004	0.09	0.79
DS (DR)	2	0.0095	17.07	<0.0001
LR	1	0.0008	0.06	0.83
LS (LR)	2	0.0279	50.26	<0.0001
T	1	0.0102	36.85	<0.0001
Error	184	0.0511		

Model I tested the effects of detritivore assemblage (D), plant litter assemblage (L), temperature (T), their interactions, and the experimental trial (nested within temperature). Model II separated the effects of detritivore assemblages into effects of species richness (DR; 1 vs. 3 species) and species identity (DS), as well as plant litter assemblages into effects of species richness (LR) and species identity (LS). Degrees of freedom, sum of squares, *F* statistic and P-values are shown.

**Table 2:** Results of general linear models testing effects of detritivore and plant litter species identity on decomposition rates (measured as the proportion of leaf weight loss per capita).

Source	df	SS	*F*	*p*	Direction of effect
**Detritivores**					
Ak	1	0.0061	12.86	0.0004	+
Lv	1	0.0035	7.27	0.0077	–
Tg	1	0.0003	0.56	0.46	No effect
Error	188				
**Plant litter**					
Ab	1	0.0100	26.33	<0.0001	–
Cl	1	0.0002	0.43	0.51	No effect
Eb	1	0.0186	49.04	<0.0001	+

Factors were the presence or absence (coded as 1 or 0) of each species: Ak, *Anisocentropus kirramus*; Lv, *Lectrides varians*; Tg, *Triplectides gonetalus*; Ab, *Apodytes brachystyllis*; Eb, *Endiandra bessaphila*; Cl, *Cryptocarya leucophylla*. Degrees of freedom, sum of squares, *F* statistic, P-values, and the direction of each effect are shown: + or − indicate, respectively, that presence of a species in a treatment resulted in faster or slower decomposition than in treatments without it.

**Figure 3:**
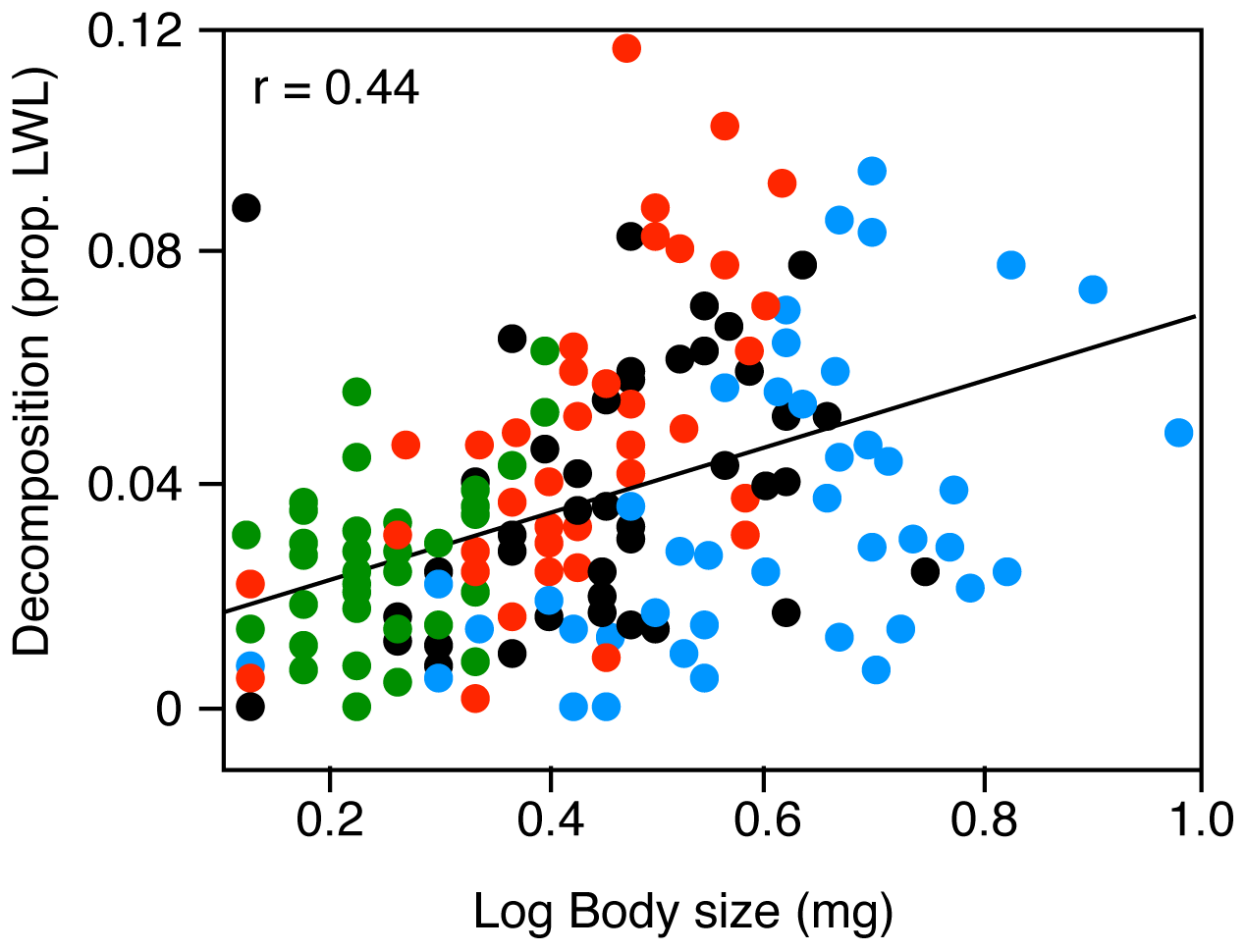
Relationship between body size and decomposition. Linear regression between detritivore body size (log dry weight of all individuals in a replicate) and decomposition rate (proportion of leaf weight loss per capita). Different colours represent different detritivore assemblages (red, *Anisocentropus kirramus* monoculture; green, *Lectrides varians* monoculture; blue, *Triplectides gonetalus* monoculture; black, polyculture).

Temperature had a significant effect on decomposition (Table 1, Figs. 1, 2). The analysis of effect sizes allowed us to compare the magnitude of temperature effects with the magnitude of species-loss effects; the loss of all species but *L. varians* had an effect as large as the effect of temperature change (Fig. 2). Decomposition rates increased with body size of the detritivores (F_1,190_ = 46.9, p<0.0001; Fig. 3), and this species trait could at least partly explain differences between the polycultures and certain monocultures. The 3 species differed in body size [F_2,141_ = 57.67; p<0.0001; mean ± SD dry weight: *T. gonetalus* (0.31±0.17 g) >*A. kirramus* (0.17±0.07) >*L. varians* (0.08±0.03)], and also in decomposition rates, although *A. kirramus* showed higher decomposition rates than *T. gonetalus* even if individuals of the former species were smaller (Fig. 1); this suggests that body size was not the only determinant of decomposition rates, and that other species traits have an influence on the efficiency of leaf litter consumers. Nevertheless, the effect of body size on decomposition was as large as the effect of temperature change (Fig. 2).

### Plant litter species loss vs. temperature

The plant litter assemblage affected decomposition (Table 1) but, when we separated species richness from species identity effects, we found that differences were due to differences between monocultures, rather than to species richness effects (Table 1, Fig. 1). Different plant litter species affected decomposition differently (Table 2), again with different polyculture-monoculture comparisons having different signs. The analysis of effect sizes also confirmed that there was no significant effect when plant litter polycultures were compared to the mean monoculture, while differences between the polycultures and some particular monocultures were significant (Fig. 2).

Where plant litter assemblages had a significant effect on decomposition, the effect was as large as the effect of temperature (*Endiandra bessaphila* monoculture vs. polyculture) or larger than it (*Apodytes bracysthyllis* monoculture vs. polyculture) (Fig. 2). SLA did not seem to explain differences between species or between monocultures and polycultures as the species that was decomposed fastest showed the lowest SLA.

## Discussion

Our experiment shows that the loss of particular detritivore and plant litter species can have significant consequences on plant litter decomposition rates. Like others, we show that species identity, rather than species richness, is a key driver of decomposition [29], [31]–[35]. More importantly, we show that the loss of particular species can equal or exceed substantial temperature change in its impact on decomposition.

Detritivore assemblages in our experiment consisted of three caddisfly species, which dominated natural assemblages. Decomposition was inhibited by the loss of certain species, and this could be partly explained by their body size, which was positively related to per capita decomposition rates. Moreover, when decomposition was corrected by detritivore biomass (rates per mg; Figure S1), the larger species that enhanced decomposition per capita (*A. kirramus*) had no effect, while the smaller species with no per capita effects (*L. varians*) enhanced decomposition rates per mg. These results suggest that, unsurprisingly, body size is a fundamental functional trait to take into account when exploring biodiversity effects of consumers on ecosystem functioning, most likely because it is a key driver of metabolic requirements [29]. Our analysis of effect sizes further showed that changes in mean detritivore body size in an assemblage can have effects on decomposition rates (either per capita or per mg) that are equal to or greater than those due to temperature changes.

The importance of detritivore body size for decomposition in a natural ecosystem, however, may be moderated by population size: for example, a large population of small shredders may consume more material than a small population of large shredders. We kept population size constant to simulate a numerical response of detritivores to species loss – i.e., when one or several species are lost, the remaining species increase in abundance [31]. Other studies have found significant density-dependent detritivore effects on decomposition [36], but the relative effects of abundance and body size on decomposition have not yet been examined. Here we examined effects of decomposition rates per capita and per mg of detritivore because they provide complementary information: the former can help explain biological mechanisms caused by differences in detritivore body size (e.g., interactions between individuals [37]), while the latter may give a more realistic quantitative estimate of what happens in the ecosystem when a species goes extinct, particularly if body size and population size are negatively related and assuming field densities are known. In both cases, we showed that effects of losing particular detritivore species were comparable to temperature effects.

Different plant species decomposed at different rates, and the consequences of losing particular species were equal or greater to those of changing temperature. Thus, certain leaf traits have a major influence on decomposition rates by detritivores, although specific leaf area (SLA) – the leaf trait that we examined – did not affect decomposition, in contrast to other studies [24]. Other leaf traits such as nutrient content or concentration of allelopathic chemicals affect leaf palatability for detritivores [38] and thus are likely to determine which plant litter species affect overall decomposition rates. Our experiment also showed that differences in mass-specific (per mg rather than per capita) decomposition rates between plant monocultures and polycultures were weaker than those between detritivore monocultures and polycultures, supporting previous findings that diversity has stronger top-down than bottom-up effects on decomposition [20].

Overall, our experiment supports previous findings about diversity effects on plant litter decomposition, especially the importance of species identity in driving decomposition rates in multi-species assemblages. But we have also shown, for the first time, that species identity effects on decomposition can equal or exceed the effects of substantial temperature change, which is often presumed to be the major environmental driver of decomposition [17]. Detection of the contrast in effects of species reduction and temperature depends partly on the chosen temperatures, as higher temperature causes faster breakdown [17]. We selected a substantial temperature change that was nevertheless within the normal tolerance range of the species tested, to avoid heat stress effects, and within credible boundaries of predicted climate change, to lend relevance to the results. Further research can identify whether the difference is a linear response to temperature change. It would also be beneficial to extend this experiment over a wider range of taxa, a wider range of systems, and greater periods of time to determine whether our results are generally applicable. Should they be so then the task of predicting effects of species loss is made more difficult as it will depend on an appropriate level of knowledge of assemblages and their constituent species' ecology and ecophysiology. This contrasts with recent findings that species richness per se has effects on decomposition that are equal or greater than effects of other environmental drivers such elevated CO_2_ and nitrogen addition [5].

## Supporting Information

Figure S1 (DOCX)
