# Supplementary material for: Biotic vs. Abiotic Control of Decomposition: A Comparison of the Effects of Simulated Extinctions and Changes in Temperature
Source: PLoS One. 2014 Jan 23;9(1):e87426. doi: 10.1371/journal.pone.0087426 (PMC3900723; doi:10.1371/journal.pone.0087426)
Supplement: Figure S1 — (DOCX) [file pone.0087426.s001.docx]

**SUPPORTING INFORMATION**

**Biotic vs. abiotic control of decomposition: a comparison of the effects of simulated extinctions and changes in temperature**

Luz Boyero, Bradley J. Cardinale, Mikis Bastian and Richard G. Pearson

**Supplementary Results**

Here we present results for decomposition rates per mg of detritivore (logit-transformed). All statistical analyses were described in the main text.

*Detritivore species loss vs. temperature*

There were significant effects of the detritivore assemblage on decomposition (Table S1). These effects were due to differences in decomposition between the different monocultures, rather than differences due to species richness per se (Table S1, Fig. S1). Different detritivore species had different effects on decomposition (Table S2): the presence of *L. varians* in a treatment resulted in faster decomposition than in treatments without it, *T. gonetalus* species had the opposite effect, and *A. kirramus* had no effect. The analysis of effect sizes showed similar results (Fig. S2). Temperature had a significant effect on decomposition (Table S1, Figs. S1, S2). In cases where detritivore assemblages had a significant effect on decomposition, this effect was as large as the effect of changing temperature (Fig. 2). Decomposition rates per mg of detritivore decreased with mean body size (F_1,190_ = 39.1, p < 0.0001; Fig. S3). Analysis of effect sizes showed that the effect of body size was as large as the effect of temperature change (Fig. S2).

*Plant litter species loss vs. temperature*

The plant litter assemblage had no effects on decomposition and, when we separated species richness from species identity effects, none of these factors were significant (Table S1, Fig. S1). *Cryptocarya leucophylla* had a negative effect on decomposition, while the other two species had no effects (Table S2). The analysis of effect sizes showed a significant difference between only the *A. brachystyllis* monoculture and the polyculture, and this effect was equal to the effect of temperature change and smaller than most effects of detritivore assemblages (Fig. S2). SLA did not explain differences between species (see main text).

**Table S1**. Results of general linear models testing the effects of detritivore and plant litter assemblages and water temperature on detritivore-mediated decomposition rates (measured as the logit-transformed, proportion of leaf weight loss per mg of detritivore). Model I tested the effects of detritivore assemblage (D), plan litter assemblage (L) and temperature (T), their interactions, and the experimental trial (nested within temperature). Model II separated the effects of detritivore assemblages into effects of species richness (DR; 1 vs. 3 species) and species identity (DS), as well as plant litter assemblages into effects of species richness (LR) and species identity (LS). Degrees of freedom, sum of squares, *F* statistic and *p*-values are shown.

| Source |  | df | SS | *F* | *p* |
| --- | --- | --- | --- | --- | --- |
| Model I |  |  |  |  |  |
| D |  | 3 | 3.23 | 6.04 | 0.0006 |
| L |  | 3 | 0.71 | 1.33 | 0.27 |
| T |  | 1 | 2.62 | 14.69 | 0.0002 |
| D x L |  | 9 | 0.95 | 0.59 | 0.80 |
| D x T |  | 3 | 0.30 | 0.56 | 0.64 |
| L x T |  | 3 | 0.73 | 1.36 | 0.27 |
| D x L x T |  | 9 | 0.44 | 0.28 | 0.98 |
| Trial |  | 4 | 0.72 | 1.00 | 0.41 |
| Error |  | 156 | 27.83 |  |  |
| Model II |  |  |  |  |  |
| DR |  | 1 | 0.005 | 0.003 | 0.96 |
| DS (DR) |  | 2 | 3.230 | 9.59 | 0.0001 |
| LR |  | 1 | 0.019 | 0.06 | 0.84 |
| LS (LR) |  | 2 | 0.693 | 2.06 | 0.13 |
| T |  | 1 | 2.622 | 15.57 | 0.0001 |
| Error |  | 184 | 30.971 |  |  |

**Table S2.** Results of general linear models testing effects of detritivore and plant litter species identity on decomposition rates (measured as the logit-transformed, proportion of leaf weight loss per mg of detritivore). Factors were the presence or absence (coded as 1 or 0) of each species. Ak, *Anisocentropus kirramus*; Lv, *Lectrides varians*; Tg, *Triplectides gonetalus*; Ab, *Apodytes brachystyllis*; Eb, *Endiandra bessaphila*; Cl, *Cryptocarya leucophylla*. Degrees of freedom, sum of squares, *F* statistic, *p*-values, and the direction of each effect are shown: + or – indicate, respectively, that presence of a species in a treatment resulted in faster or slower decomposition than in treatments without it.

| Source | df | SS | *F* | *p* | Direction of effect |
| --- | --- | --- | --- | --- | --- |
| Detritivores |  |  |  |  |  |
| Ak | 1 | 0.18 | 0.99 | 0.32 | No effect |
| Lv | 1 | 1.15 | 6.29 | 0.013 | + |
| Tg | 1 | 1.91 | 10.44 | 0.0015 | – |
| Error | 188 |  |  |  |  |
| Plant litter |  |  |  |  |  |
| Ab | 1 | 0.04 | 0.22 | 0.64 | No effect |
| Eb | 1 | 0.10 | 0.49 | 0.48 | No effect |
| Cl | 1 | 0.57 | 2.93 | 0.089 | – |
| Error | 188 |  |  |  |  |

**Fig. S1**. Mean ± SE detritivore-mediated decomposition rates (measured as the logit-transformed, proportion of leaf weight loss per mg of detritivore) in each detritivore/plant litter assemblage and temperature treatment. Different letters within panels indicate significant differences. Ak, *Anisocentropus kirramus*; Lv, *Lectrides varians*; Tg, *Triplectides gonetalus*; Ab, *Apodytes brachystyllis*; Eb, *Endiandra bessaphila*; Cl, *Cryptocarya leucophylla*; PC, polyculture.

**Fig. S2.** Effect sizes (Cohen’s d and 95% confidence interval) of detritivore and plant litter assemblages (each monoculture compared to the polyculture; black, solid bars) and detritivore body size (black, broken bars) compared to temperature (grey shade), on detritivore-mediated decomposition rates (measured as the logit-transformed, proportion of leaf weight loss per mg of detritivore). Effects sizes for body size were obtained by converting the correlation index *r* derived from linear regression analysis (Fig. 3) to Cohen’s *d* using the formula: *d* = 2*r* / √1-*r*^2^ (see main text). Only significant effects are shown. B. size, detritivore body size; Lv, *Lectrides varians*; Tg, *Triplectides gonetalus*; Ab, *Apodytes brachystyllis*.

**Fig. S3**. Linear regression between log-transformed detritivore body size (measured as dry weight of all individuals in a replicate) and decomposition rates (measured as the logit-transformed, proportion of leaf weight loss per mg of detritivore). Different colours represent different detritivore assemblages (red, *Anisocentropus kirramus* monoculture; green, *Lectrides varians* monoculture; blue, *Triplectides gonetalus* monoculture; black, polyculture).
